# Five-Year Status of Malaria (a Disease Causing Anemia) in Yazd, 2008-2012

**Published:** 2013-07-22

**Authors:** A Fattahi Bafghi, S A Pourmazar, F Shamsi

**Affiliations:** 1 Associate Professor, Medical Parasitology & Mycolology Department. The School of Medicine, Shahid Sadoughi University of Medical Sciences and Health Services, Yazd, Iran.; 2 GP & Head unit of Control Contagious Diseases, Health center of Yazd province, Shahid Sadoughi University of Medical Sciences, Yazd, Iran.; 3 MSc in Biostatics, Biostatic and Epidemiology Department, The School of Heath, Shahid Sadoughi University of Medical Sciences and Health Services, Yazd, Iran.

**Keywords:** Malaria, Anemia, Plasmodium falciparum, Iran

## Abstract

**Background:**

Yazd province which is the host of local and foreign immigrants may be faced with contacting malaria. Plasmodium falciparum malaria remains a major cause of mortality throughout in the tropical regions of the world. Pthophysiologic mechanisms of anemia in malaria is such as direct invasion of Red cells, anemia of chronic disease hypersplenism, Hemophagocytic syndrome and erythrophagocytosis, dyserythropoirsis, immune haemolysis and cytokine deregulation anemia of chronic disorder is characterized by moderate to mild normocytic, normochromic anemia along with microcytic hypochromic cells. Malaria occurs predominantly in children in the first three years of life. The purpose of this study was Demographic study of malaria during 2008 to 2012 in Yazd**.**

**Materials and Methods:**

This study was an analytic-descriptive and manner descriptive study. All episodes (Imported Malaria) of disease from 2008 to 2012 which were documented in Yazd Central Health Service were carefully studied and reported.

**Results:**

A total of 206 confirmed reported malaria patients from 2008 to 2012 were studied; Plasmodium (P). vivax species was mostly, 187(90.78%) and Plasmodium (P). Malaria species was Lesley, 1(0.49%). The mean age groups, accommodation with local malaria and years of reported outbreaks of different strains of the parasite conducted by Fisher exact Test, showed no significant difference(P-value>0.05), but the mean of foreigner immigrants of outbreaks of different strains of the parasite conducted by Fisher exact Test, showed significant difference (P-value= 0.01).

**Conclusion:**

Although malaria has been designed on elimination program in Iran, but in the province of Yazd is reported imported malaria and its importance in causing anemia and other blood disorders is not negligible.

## Introduction

Anemia is a condition that develops when the blood does not contain enough healthy red blood cells. These cells are the main transporters of oxygen to the organs in the body. Symptoms of anemia - like fatigue - occur because the organs aren't getting enough oxygen. There are many types of anemia. All Of them are very different in their causes and treatments. Iron-deficiency anemia, the most common type, is easily treated with dietary changes and iron supplements. However, some types of anemia may present lifelong Failure of health. There are more than 400 types of anemia, which are divided into three groups: Red blood cells can be lost through bleeding, which can occur slowly over a long period of time, and can often go undetected, decreased or faulty red blood cell production The body may produce few blood cells or the blood cells may not work properly. In either case, could lead to anemia. Red blood cells may be decreased due to abnormal red blood cells or a lack of minerals and vitamins needed for red blood cells to work, properly and excessive destruction of red blood cells (1-3). 

Malaria has been recognized as a severe and life-threatening illness for thousands of years. It still is one of the most common diseases affecting humans worldwide. The major impact of the disease is almost nearly on the developing countries, with the heaviest burden in Africa. Almost half of the total world population is exposed to the risk of contacting malaria. To summarize, malaria is the leading cause of death and disease in many developing countries. According to the World Health Organization’s World Malaria Report 2011 and the Global Malaria Action Plan, 3.3 billion people worldwide live in areas at risk of malaria transmission in 106 countries and territories. In 2012 malaria led to 216 million clinical episodes, and 655,000 deaths. An estimated 91% of deaths in 2010 were in the African region, followed by 6% in the south-east Asian region and 3% in the eastern Mediterranean region (3%). 86% of all deaths worldwide are children (4).

Over 40% of the world’s populations are at risk of contacting malaria, which is endemic in 91 countries, mostly developing countries. Malaria has long featured prominently in the grey area between parasitological and hematological. Despite of intensive worldwide efforts to reduce its transmission, malaria remains the most serious and widespread protozoan infection of humans. In a classical European textbook of hematology published in the 1930s (5,6). Malaria was defined as a “typical blood disease” characterized by fever, anemia and splenomegaly. Due to an acquired extra-corpuscular cause, it is currently considered a typical example of a hemolytic anemia in more recent hematology textbooks. As parasites of blood for the majority of their complex life cycle, they expectedly induce hematological alterations. The hematological abnormalities that have been reported to invariably accompany infection with malaria include anemia, thrombocytopenia, splenomegaly, and mild-to-moderate atypical lymphocytosis and rarely disseminated intravascular coagulation (DIC). There have also been reports of leukopenia and leukocytosis. Other hematological reactions to malaria that have been reported include neutropenia, eosinophilia, neutrophilia and monocytosis.

Iran is divided into two district malaria: a. Malaria-prone areas included Sistan and Baluchistan, Hormozgan and Kerman in south of Iran and b. imported malaria areas; Imported malaria and the origin is domestic (return of malaria- prone areas of Iran) or foreign origin. Yazd province which is the host of local and foreign immigrants may be faced contacting malaria. Plasmodium falciparum malaria remains a major cause of mortality throughout the tropical world. Hematological abnormalities are considered a hallmark of malaria, bearing an impact on final outcome and representing indices of prognostic and follow-up value, which include severe anemia, coagulation disturbances, leukocyte numerical or functional changes and spleen involvement. Anemia is a common complication in malarial infection, although the consequences are more pronounced with *Plasmodium falciparum*. Anemia in this infection is caused by a variety of patho physiologic mechanisms, and in areas where malaria infection is endemic, co-morbidities like other parasitic infestations, iron, foliate and vitamin B12 deficiency, deficiency of other nutrients, and anemia, which is aggravated by anti-malarial drugs both through immune and non-immune mechanisms, is important considerations. In different endemic areas, β-thalassemia, α-thalassemia, Hb S, Hb E, G6PD deficiency, or ovalocytosis in different proportions interact with this infection. Finally, aberrant immune response to repeated or chronic falciparum malarial infection may produce tropical splenomegaly syndrome, a proportion of which show clone proliferation of B lymphocytes. Cooperation between chronic malarial infection and infection with E-B virus infection in producing Burritt's lymphoma is well known. Anemia involves red blood cell lyses due to parasite invasion, as well as mechanisms of intravascular hemolysis and decreased erythropoiesis. Exchange or blood transfusion is mainly recommended in the management of these patients. Hemorrhagic complications in severe malaria are relatively rare despite prominent thrombocytopenia and dysfunction in the coagulation pathway. Numerical, as well as functional changes in the white blood cell are less dramatic than other blood cell series, but still, remain a significant index of disease progression and ultimate prognosis. The province of Yazd is located exactly at the center of Iran, closed to two endemic provinces of Kerman and Sistan and Baluchistan; where generate 79% of total malaria cases in Iran. Therefore imported malaria incidence in this area is imported and is due to the presence of workers come to Yazd for work mostly as construction workers. The purpose of this study was Demographic study of malaria during 2008 to 2012 in Yazd (9-17).

## Materials and Methods

The study has been approved by the Ethic Committee of Shahid Sadoughi University of Medical Sciences, Yazd, Iran. The present descriptive (study retrospective) was conducted to indicate epidemiological feature of Malaria in the province of Yazd, Iran. Therefore, all episodes from 2008 to 2012 documented in Yazd Central Health Service (CHS) was carefully studied and reported. Malarial patients were Iranian and foreigner people but all had resided in Yazd. In general, all suspected malarial patients are referred to central health service located in the city of Yazd and checked by physicians working in different clinics or hospital. 

**Statistical Analysis**

The data was analyzed using SPSS Version 17 statistical software. Chi-square test was used for data analysis of qualitative variables, and values were compared using independent Fisher- exact Test. Differences were considered significant at P-values of less than 0.05. Informed consent was taken from patients and parents. 

## Results

Data showed that 90.78% of isolated species was P (P). Vivax, 7.28% species was Plasmodium (Lavrania).falciparum, 0.1% P (P). Malariae and 1.45% were mixed species. The mean age groups, accommodation with local malaria and years of reported outbreaks of different strains of the parasite conducted by Fisher exact Test, showed no significant difference (P-value> 0.05) (Table I). 179 (86.89 %) of malarial patients were male and 27 (13.11 %) of them were female. The Mostly malarial patients were foreigner immigrant (48.54 % of patients were Afghan, 12.14 % Pakistani and 3.9% Indian and other nationalities). The mean of different strains outbreak of the parasite among foreigner immigrants conducted by Fisher exact Test, showed significant difference (P-value= 0.01) (Table II). A few of malarial patients were Iranian (14.1 % of patients were from Sistan and Baluchistanian, 9.7 % Yazd and 4.9 % Kerman) (Table III).

**Table I:** Prevalence of imported malaria according to age groups

**Total**	**Mixed Species**	**P(P)malariae**	**P(L).falciparum**	**P(P).vivax**	**Age Groups**
8 100.0%	0 .%	0 .0%	1 12.5%	7 87.5%	**1- 10**
50 100.0%	2 4.0%	1 2.0%	2 4.0%	45 90.0%	**11- 20**
79 100.0%	1 1.3%	0 .0%	8 10.1%	70 88.0%	**21- 30**
38 100.0%	0 0%.	0 .0%	3 7.9%	35 92.1%	**31- 40**
31 100.0%	0 .0%	0 0%.	1 3.3%	30 90.8%	**41>**
20 100.0%	3 1.5%	1 0.5%	15 7.3%	18 90.8%	**Total**

*Fisher exact – Test. P-value>0.05*

**Table II.** Prevalence of imported malaria according to Nationality

**Total**	**Mixed Species**	**P(L).falciparum**	**P(P).vivax**	**Reported Years**
100 100.0 %	1 1.4%	6 6.0 %	93 93.0 %	**Afghanistan**
25 100.0 %	1 4.0 %	1 4.0 %	23 92.0 %	**Pakistan**
8 100.0%	0 .0 %	3 37.5 %	5 62.5%	**India**
133 100.0%	2 1.5%	10 7.5%	121 91.0%	**Total**

*Fisher exact – Test. P-value=0.001*

**Table III:** Prevalence of imported malaria according to the origin Iranian patients

**Total**	**Mixed Species**	**P(P)malariae**	**P(P).falciparum**	**P(P).vivax**	**Reported Years**
20 100.0%	0 .0 %	0 .0 %	3 15.0 %	17 85.0%	**Yazd**
54 100.0%	1 1.85%	1 1.85%	2 3.7%	50 92.59%	**Malaria-prone areas**
74 100.0%	1 1.4 %	1 1.4 %	5 6.8 %	67 90.5 %	**Total**

*Fisher exact – Test.P-value>0.05.*

**Figure 1:**
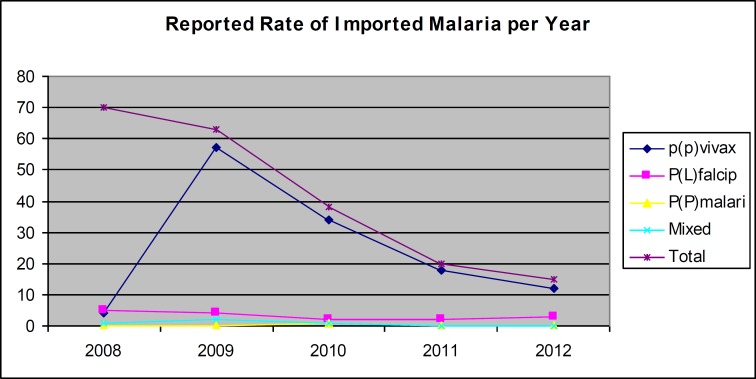
Reported rate of imported malaria species per year. Fisher exact – Test. P-value>0.05

## Discussion

Malarial anemia is an enormous public health problem in endemic areas and occurs predominantly in children in the first 3 years of life. Anemia is due to both a great increase in clearance of uninfected cells and a failure of an adequate bone marrow response. Iran is among the countries located in the Middle East with low malaria endemicity and in its some areas, there is the risk of malaria transmission**. **Yazd province which is the host of local and foreign immigrants may be faced contacting malaria. Imported malaria is limited in Yazd but since the province is highly active in industry and construction projects, it has become the host of thousands of male refuges from the endemic malarial areas of Afghanistan and south-eastern of Iran. 

Although imported malaria does not depend on the age groups, but in this study, like most previous studies, the active age groups showed the highest rates of infection to imported malaria, and this is because being more susceptible to disease reservoirs and vectors (18). Like all previous studies, the highest number of malaria for Afghan refugees in Iran and although the planning currently in minister of health is to elimination of malaria, the greatest potential risk of disease entering of patients from the country's eastern borders(19, 20). There is a risk of disease incidence. Although eradication program has failed, however elimination program of the disease may also be an efficient program. The most cases of imported malaria in Yazd, is related to foreign immigrants, but Yardman farmers drivers and the other travelers carry a high statistics of imported malaria (21, 22, 23, 24). More attentions are needed to traffic control, passengers, especially foreigners, to enable health centers and placing courses malaria experts in health centers, health education to the public, especially those at risk, prognosis and treatment of patients, including measures necessary to control imported malaria in Yazd. Since different reports revealed increasing resistance of malarial species against anti-malarial drugs, therefore the best suggestion could be providing free and enough health facility for suspected patients to come to health service for treatment. In addition, this is the task of health service workers to trace up the patients until complete treatment achieved. Although malaria has been designed on elimination program in the country, but in the province of Yazd is reported as immigration (Imported Malaria) and its importance in causing anemia and other blood disorders is not negligible. 

## Conclusion

Based on the result of this study, although in the province of Yazd malaria is reported as immigration (Imported Malaria) but its importance in causing anemia and other blood disorders is not negligible, Physicians, especially those in malaria- prone areas,

Should be aware of the varied manifestations and maintain a high index of suspicion for the disease so that the diagnosis and treatment are timely and morbidity and mortality minimized.
